# LIN28B inhibition sensitizes cells to p53-restoring PPI therapy through unleashed translational suppression

**DOI:** 10.1038/s41389-022-00412-8

**Published:** 2022-07-02

**Authors:** Jiahao Shi, Xiaoliang Jin, Yihao Wang, Tianyu Zhu, Dongmei Zhang, Qian Li, Xiaomin Zhong, Yaqi Deng, Jianfeng Shen, Xianqun Fan

**Affiliations:** 1 Department of Ophthalmology, Ninth People’s Hospital, Shanghai Jiao Tong University School of Medicine, 200025 Shanghai, China; 2 Shanghai Key Laboratory of Orbital Diseases and Ocular Oncology, 200025 Shanghai, China; 3 Department of Obstetrics and Gynecology, Reproductive Medical Center, West China Second University Hospital, Sichuan University, 610041 Chengdu, China; 4 Institute of Translational Medicine, National Facility for Translational Medicine, Shanghai Jiao Tong University, 200240 Shanghai, China; 5 Key Laboratory for Stem Cells and Tissue Engineering, Ministry of Education, Center for Stem Cell Biology and Tissue Engineering, Zhongshan School of Medicine, Sun Yat-Sen University, 510080 Guangzhou, China

**Keywords:** Cancer, Oncogenes, Cancer therapy

## Abstract

p53 is the most highly mutated tumor suppressor across multiple types of human cancers. The level and function of p53 are fine-tuned through multifaced mechanisms in which the protein–protein interaction between p53 and MDM2 is considered as a major circuit. Recent studies suggest therapeutic strategy attempts to restore p53 function by small molecule inhibitors targeting p53–MDM2 interaction can be a promising direction in treating cancers with wild-type or functional p53. Currently, clinical tests of the p53–MDM2 protein–protein interaction inhibitors (PPIs) are underway. However, it remains elusive about the biomarkers that may predict the therapeutic responses to those inhibitors. Here we report that RNA-binding protein LIN28B directly regulates p53 through binding to the 5′΄ untranslated region of p53 mRNA and blocks its translation by competing with a translation enhancer protein, ribosomal protein L26 (RPL26). This regulatory mechanism of LIN28B does not involve let-7 maturation or the canonical protein turnover pathway of p53. Furthermore, we show that inhibition of LIN28B unleashes the translational suppression of p53 through RPL26, and leads to enhanced sensitivities of cancer cells to inhibitors of p53–MDM2 interaction. Together, we demonstrate a competitive regulatory mechanism of p53 by LIN28B, which has important implications in developing biomarkers to the therapies aiming to reinstate p53 function.

## Introduction

p53 (encoded by the tumor suppressor gene *TP53*) is a genome guardian that has a broad impact on DNA damage, cell cycle arrest, and apoptosis [1–3]. Accordingly, *TP53* gene mutation or loss is the most common genetic lesion in human cancer, occurring in nearly 50% of all cancer types, and aberration of p53 function is even more frequently observed [4, 5]. In addition to abolishing the tumor suppressor function, p53 mutations also endow the mutant p53 proteins the ability to contribute to tumor progression and drug resistance [6]. The regulations of p53 level and function are highly orchestrated, for which the MDM2/4-mediated polyubiquitination and proteasomal degradation of p53 is the key mechanism [7–9]. MDM2/4 not only affects p53 protein degradation but also suppresses the transcriptional function of p53 by interfering with its association with co-activators [10]. Thus, the imbalance of the p53–MDM2/4 interaction is considered a critical mechanism that contributes to tumorigenesis [11].

Recent studies show that strategies to reinstate p53 function by targeting the interaction between p53 and MDM2 are promising in treating cancers harboring wild-type or functional copy of *TP53* [12]. Therefore, protein–protein interaction inhibitors (PPIs) antagonizing the p53–MDM2 loop have been developed [13–15], and their therapeutic efficacies are currently under evaluation in clinical trials (*ClinicalTrials.gov*). Despite previous reports have suggested that MYCN-amplification may render neuroblastoma cells sensitive to p53–MDM2 PPIs [16, 17], little is known about the mechanistic details and whether other factors may serve as biomarker to predict the therapeutic responses to those PPIs, especially in cancers without MYCN-amplification. To address this, further elucidation of the regulatory mechanisms of p53 is demanded.

p53 function is also regulated at translational levels. Several factors interact with *TP53* mRNA at either the 3′ or the 5’ untranslated region (UTR) to influence p53 protein translation, including human antigen R (HuR) and ribosomal protein L26 (RPL26) [18]. HuR acts on the 3′UTR of *TP53* mRNA and increases p53 protein stability and level [19]. In contrast, binding of RPL26 to the 5′UTR of *TP53* mRNA enhances p53 translation, leading to increased p53 protein level [20–22]. We previously demonstrated that LIN28B promotes tumor growth and inhibits apoptosis by modulating the AKT2/FOXO3A/BIM axis [23]. This finding implies a plausible link between p53 signaling and the tumorigenic function of LIN28B, an evolutionarily conserved key player that orchestrates multiple physiological and pathological processes including development and cancer [24–27]. The abnormal re-activation of LIN28B is commonly seen in diverse human malignancies and serves as an oncoprotein by modulating tumor progression, metastasis and cancer immunity [1, 28–31]. However, whether and how LIN28B may directly regulate p53 is unknown.

In the present study, we demonstrate that the LIN28B downregulates p53 protein through a translational repression mechanism rather than at the mRNA level, which involves the competition with RPL26 to bind at the 5′UTR of *TP53* mRNA. Importantly, LIN28B inhibition unleashes the translational suppression of p53 through RPL26, and leads to enhanced sensitivities to p53–MDM2 PPIs in cancer cells. These findings of p53 regulation by LIN28B provide insights into how cancer cells can impede the function of a key tumor suppressor in the absence of gene mutation, and potentiate the application of LIN28B as a stratification biomarker to p53–MDM2 PPI therapy.

## Results

### LIN28B inhibition enhances p53 activity

**Fig. 1:**
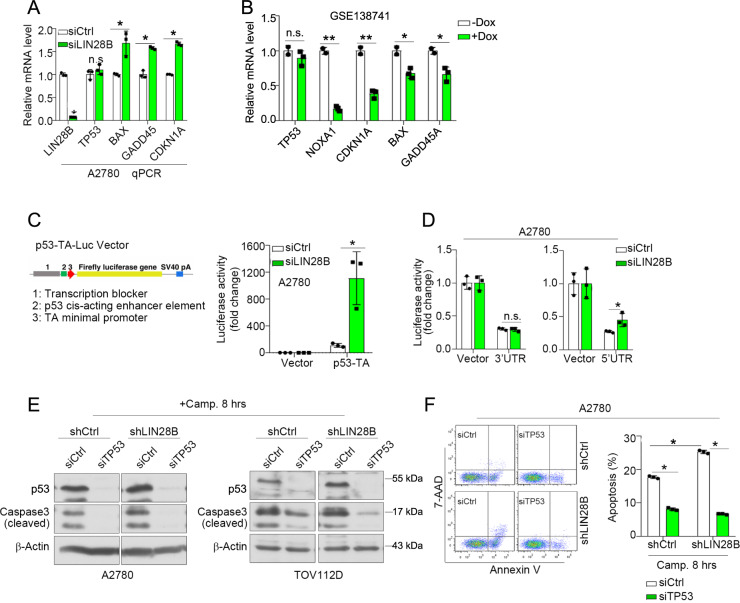
LIN28B inhibition enhances p53 activity. **A** qPCR of *LIN28B*, *TP53*, *BAX*, *GADD45*, and *CDKN1A* in control (si*Ctrl*) and LIN28B knockdown (si*LIN28B*) A2780 cells. Representative data of three independent experiments (mean ± s.e.m.). *p < 0.05. n.s. = not significant. **B** Relative mRNA levels of *TP53*, NOXA1, CDKN1A, *BAX,* and *GADD45* in control (−Dox) and LIN28B-induced (+Dox) BE2C neuroblastoma cells by analyzing the GSE138741 dataset [47]. *p < 0.05, **p < 0.01. n.s. = not significant. **C** p53 luciferase activity (pTA-p53) in control (si*Ctrl*) and LIN28B knockdown (si*LIN28B*) A2780 cells. Representative data of three independent experiments (mean ± s.e.m.). *p < 0.05. **D** Luciferase activity in control (si*Ctrl*) and LIN28B knockdown (si*LIN28B*) A2780 cells utilizing *TP53* 3′UTR (pmirGLO) or 5′UTR (pGL3) reporters. Representative data of three independent experiments (mean ± s.e.m.). *p < 0.05. n.s. = not significant. **E** Representative western blots of p53 and cleaved caspase-3 in control (si*Ctrl*) and p53 knockdown (si*TP53*) cells under camptothecin (2 μM, 8 h) treatment. **F** FACS analysis of apoptosis (Annexin V labeling) in control (si*Ctrl*) and p53 knockdown (si*TP53*) cells under camptothecin (2 μM, 8 h) treatment. Representative data of three independent experiments (mean ± s.e.m.). *p < 0.05.

LIN28B was activated in multiple types of cancer cell lines (Supplementary Fig. 1A). Consistent to previous findings, knockdown of LIN28B attenuated the viability of ocular melanoma and ovarian cancer cells (Supplementary Fig. 1B–D). Importantly, put-back of LIN28B in LIN28B-depleted A2780 using CRISPR-Cas9 method (sgLIN28B) significantly increased the cell viability as compared with Vector control (Supplementary Fig. 1E). We next evaluated the levels of well-characterized p53 downstream targets, *BAX*, *GADD45*, and *CDKN1A*, as well as the activity of p53. LIN28B knockdown elevated the expression of these genes without affecting the mRNA level of *TP53* (Fig. 1A). Conversely, the induction of LIN28B markedly suppressed the levels of p53 downstream genes (Fig. 1B). These results suggested a functional modulation of p53 signaling by LIN28B. We next used a luciferase reporter system that monitors p53-binding activity as a functional assessment [32]. We found that LIN28B knockdown markedly elevated the p53-binding activity (Fig. 1C). In addition, assays using luciferase reporters driven by the 3′UTR or 5′UTR of *TP53* mRNA demonstrated that LIN28B-mediated repression of p53 protein was mainly through the 5′UTR but not the 3′UTR (Fig. 1D). In addition, we knocked down *TP53* by siRNA in both control and LIN28B-depleted cells to assess the role of p53 in LIN28B-mediated apoptosis induced by the DNA-damaging agent camptothecin. LIN28B knockdown resulted in a marked increase of apoptosis; however, these effects were significantly blocked by p53 depletion (Fig. 1E, F), indicating a critical epistatic function of p53 in LIN28B-mediated apoptosis. Without camptothecin treatment, only minor induction of apoptosis was observed as indicated by cleaved caspase3 with long exposure in western blots (Supplementary Fig. 1F) and <5% of apoptotic cells in FACS analysis (Supplementary Fig. 1G).

### LIN28B suppresses p53 protein expression

**Fig. 2:**
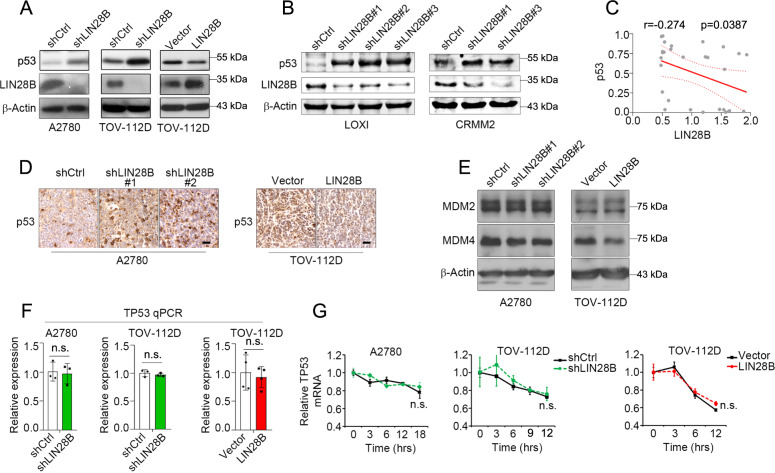
LIN28B inhibits p53 protein but not mRNA level independent of MDM2/4. **A** Representative western blots of p53 and LIN28B expression in A2780 and TOV-112D cells with LIN28B knockdown (sh*LIN28B*) and overexpression (LIN28B) from three independent experiments. **B** Representative western blots of p53 and LIN28B expression in LOXI and CRMM2 cells with LIN28B knockdown (sh*LIN28B*) from three independent experiments. **C** Correlation of LIN28B and p53 protein expression in ovarian cancer cell lines (*n* = 29) based on Supplementary Fig. 3 and our previous publication [23]. **D** Representative immunohistochemistry staining image of p53 in LIN28B knockdown (sh*LIN28B*) and overexpression (LIN28B) xenograft tumors. Bar, 100 μm. **E** Representative western blots of MDM2 and MDM4 expression in A2780 and TOV-112D cells with LIN28B knockdown (sh*LIN28B*) and overexpression (LIN28B) from three independent experiments. **F** qPCR of *TP53* mRNA level in LIN28B knockdown (sh*LIN28B*) and overexpression (LIN28B) samples. Ctrl = control. Representative data of three independent experiments (mean ± s.e.m.). n.s. = not significant. **G**, qPCR of *TP53* mRNA in LIN28B knockdown (sh*LIN28B*) and overexpression (LIN28B) cells treated by actinomycin D (10 μg/ml). Representative data of three independent experiments (mean ± s.e.m.). n.s. = not significant.

Since *TP53* mRNA appeared to be intact by LIN28B perturbation, we then tested the protein level of p53 to gain further insights into the regulatory mechanisms of LIN28B upon p53 signaling. We found that LIN28B knockdown significantly increased p53 protein levels, while LIN28B overexpression led to a reduction of p53 protein expression (Fig. 2A, B and Supplementary Fig. 2A–E). We also evaluated the protein expression of p53 in a set of cancer cell lines (Supplementary Figs. 1A and 3). Using the LIN28B expression-profiling data [23], we observed a negative correlation between p53 and LIN28B protein expression (Fig. 2C). Similar trend of correlation was found in melanoma cell lines (Supplementary Fig. 1A). Immunohistochemistry (IHC) staining of p53 in A2780 and TOV-112D xenograft tumors further supported the observations in cell lines (Fig. 2D). The p53 protein level was commonly modulated through the MDM2/4-mediated degradation [7–9], nevertheless the levels of MDM2 and MDM4 remained unchanged by LIN28B knockdown or overexpression (Fig. 2E). Importantly, MDM4 knockdown by two independent siRNA sets did not further change the level of p53 protein that was already regulated by LIN28B (Supplementary Fig. 4). These results suggest that LIN28B regulates p53 protein through mechanisms that are largely independent of protein turnover. In addition, neither knockdown nor overexpression of LIN28B altered the levels and decay rates of *TP53* mRNA (Fig. 2F, G), supporting the notion that LIN28B mostly inhibited the protein rather than mRNA level of p53.

### LIN28B inhibits p53 protein translation

**Fig. 3:**
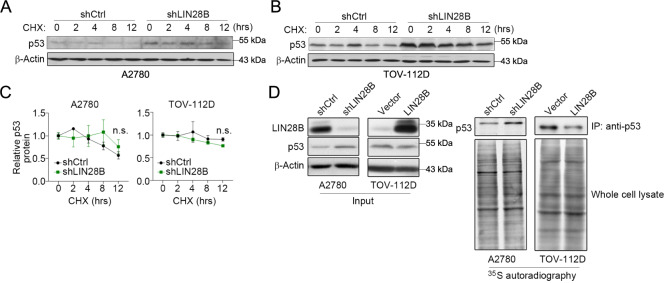
LIN28B suppresses p53 protein translation. **A** Representative western blots of p53 in control (sh*Ctrl*) and LIN28B knockdown (sh*LIN28B*) A2780 cells treated with cycloheximide (CHX, 100 μg/ml). **B** Representative western blots of p53 in control (sh*Ctrl*) and LIN28B knockdown (sh*LIN28B*) TOV-112D cells treated with cycloheximide (CHX, 100 μg/ml). **C** Quantification of p53 protein levels in (**A**) and (**B**). Representative data of three independent experiments (mean ± s.e.m.). n.s. = not significant. **D** Representative images of western blots (left) and ^35^S methionine labeling and autoradiography (right) in the indicated cells with LIN28B knockdown (shLIN28B) and overexpression (LIN28B). n.s. = not significant.

LIN28B knockdown did not alter p53 protein stability under cycloheximide treatment (Fig. 3A–C). To test whether LIN28B directly regulated p53 translation, we conducted experiments using newly synthesized p53 protein labeled by pulsing with [^35^S] methionine. We found that the overall labeling efficiency was comparable among samples. Nevertheless, LIN28B knockdown resulted in a marked increase of newly synthesized p53 protein, while overexpression of LIN28B led to a significant reduction of p53 protein synthesis (Fig. 3D). Given the let-7 circuit involved in the biological functions of LIN28B, we assessed whether this mechanism played a role in the regulation of p53. We overexpressed let-7b by miRNA mimic transfection in A2780 cells, a low let-7b expressing cell line. Indeed, let-7b mimic significantly augmented caspase-3/7 activity under camptothecin treatment (Supplementary Fig. 5A), and inhibited the expression of *HMGA2*, a well-known target of let-7b (Supplementary Fig. 5B). But the p53 protein level was not altered by let-7b mimic (Supplementary Fig. 5C). These results reflected the let-7-independent mechanisms through which LIN28B inhibited p53.

### LIN28B interacts with the 5′UTR of *TP53* mRNA

**Fig. 4:**
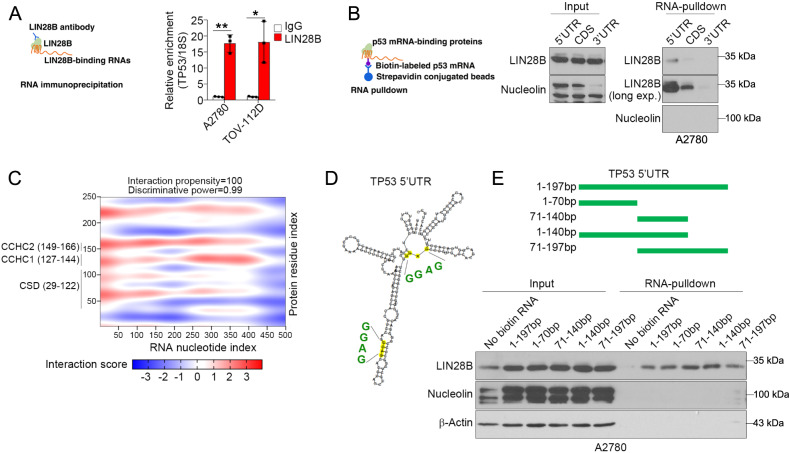
LIN28B interacts with *TP53* mRNA at the 5′ untranslated region (UTR). **A** Schematic diagram of RNA immunoprecipitation (RNA-IP) and qPCR detection of *TP53* mRNA enrichment in A2780 and TOV-112D cells using isotype control IgG or anti-LIN28B antibody. Representative data of three independent experiments (mean ± s.e.m.). *p < 0.05, **p < 0.01. **B** Schematic of RNA-pulldown and representative western blots of LIN28B and nucleolin in input and RNA-pulldown samples. CDS coding sequence. **C** Representative interaction heatmap between *TP53* mRNA (5′UTR) and LIN28B protein by catRAPID prediction. **D** Secondary structure of *TP53* mRNA (5′UTR) by RNAfold prediction. **E** Schematic diagram of truncation mutations of *TP53* 5′UTR and representative western blots of LIN28B, nucleolin and β-actin in input and RNA-pulldown samples. Exp. = exposure time. *p < 0.05.

By analyzing our previous RNA-IP data, we identified *TP53* mRNA as one of the binding targets of LIN28B as shown by RNA-IP and real-time PCR (Fig. 4A). The finding was validated by a publicly available LIN28B-enhanced CLIP sequencing (eCLIP-seq) dataset performed in 293T (GSE178259, Supplementary Fig. 6) [33]. To dissect the region of *TP53* mRNA that interacts with LIN28B, we synthesized biotin-labeled oligos of *TP53* 5′UTR, the coding sequence (CDS), and 3′UTR. RNA pulldown assay showed that LIN28B preferentially bound to the *TP53* 5′UTR rather than the 3′UTR and CDS (Fig. 4B). Consistent with these results, the prediction of protein–RNA interactions using the catRAPID bioinformatic tool suggested that LIN28B interacts with *TP53* mRNA at the 5′UTR and CDS (1–500 bp) and the 5′UTR was the preferred region (Fig. 4C). Examination into the secondary structure of *TP53* 5′UTR revealed two ‘GGAG’ sequences (Fig. 4D), which were reported as conserved LIN28B-binding motifs in both miRNAs and mRNAs [34]. We next generated various *TP53* 5′UTR deletion mutants to perform RNA pulldown analyses and to determine the minimal sequence for LIN28B binding. Consistent with the results in Fig. 4C and D, we found that both 1–70 and 71–140 bp regions were sufficient to interact with LIN28B (Fig. 4E). Of note, we did not observe the binding of nucleolin to *TP53* 5′UTR. Together, these findings showed that the 5′UTR of *TP53* mRNA was the major binding region for LIN28B and further supported the results observed in Fig. 1D.

### LIN28B suppresses p53 translation by competing with RPL26 at the 5′UTR of *TP53* mRNA

**Fig. 5:**
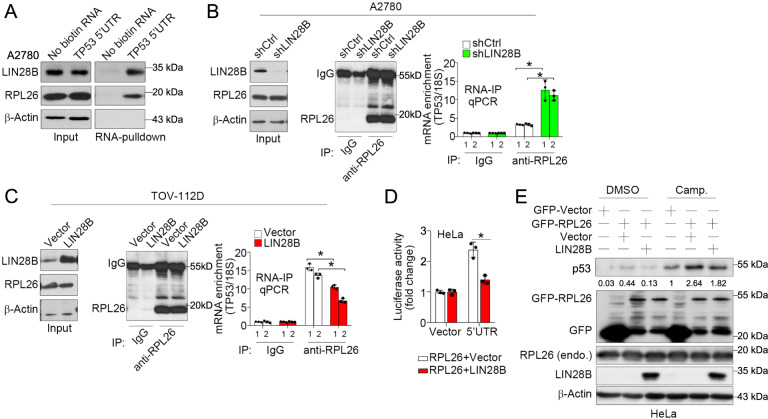
LIN28B competes with RPL26 at the *TP53* mRNA 5′UTR and inhibits its translation enhancer function. **A** Representative western blot of LIN28B and RPL26 in A2780 cells with RNA pulldown using control RNA (no biotin) and biotin-labeled *TP53* 5′UTR sequence. **B** Representative western blots of RPL26 immunoprecipitation in control (sh*Ctrl*) and knockdown (sh*LIN28B*) A2780 cells (left). qPCR of *TP53* mRNA after RNA-IP using isotype control IgG or anti-RPL26 antibody in control (sh*Ctrl*) and knockdown (sh*LIN28B*) A2780 cells (right). 1, *TP53* 5′UTR primer #1; 2, *TP53* 5′UTR primer #2. 18 S served as an internal control. Representative data of three independent experiments (mean ± s.e.m.). *p < 0.05. **C** Representative western blots of RPL26 immunoprecipitation in TOV-112D cells transfected with vector control or with LIN28B overexpression (left). qPCR of *TP53* mRNA after RNA-IP using isotype control IgG or anti-RPL26 antibody in TOV-112D cells transfected with vector control or with LIN28B overexpression (right). 1, *TP53* 5′UTR primer #1; 2, *TP53* 5′UTR primer #2. 18S served as an internal control. Representative data of three independent experiments (mean ± s.e.m.). *p < 0.05. **D** Luciferase activity of the *TP53* 5′UTR reporter with RPL26 and/or LIN28B overexpression in HeLa cells. Representative data of three independent experiments (mean ± s.e.m.). *p < 0.05. **E** Representative western blots of p53, RPL26, and LIN28B in RPL26 and/or LIN28B-overexpressing HeLa cells. Camp. camptothecin (2 μM, 8 h); endo. endogenous. Listed numbers represent the relative expression of p53.

To delineate the underlying mechanism linking LIN28B to p53 translation, we examined the 5′UTR of *TP53* mRNA. The 5′UTR of *TP53* is a highly regulated region that binds a number of translation activators or suppressors [18]. We focused on RPL26 (Ribosomal Protein L26), since it binds to the 5′UTR and enhances the association of *TP53* mRNA with heavy polysomes to increase p53 protein translation [20–22]. We examined whether LIN28B could compete with RPL26 binding to *TP53* mRNA and consequently inhibit the translation enhancer function of RPL26. We conducted RNA pulldown assays and confirmed the binding of both LIN28B and RPL26 to the 5′UTR of *TP53* mRNA (Fig. 5A). We then examined RPL26 expression in LIN28B knockdown or overexpression cells and found that LIN28B did not alter the level of RPL26 or the immunoprecipitation efficiency of anti-RPL26 antibody (Fig. 5B, C). We performed RNA-IP in LIN28B knockdown A2780 cells and LIN28B overexpression TOV-112D cells. RPL26-bound *TP53* mRNA was immunoprecipitated using an anti-RPL26 antibody and quantified by two independent real-time PCR primer sets located in the 5′UTR of *TP53* mRNA. We found that RPL26-bound *TP53* mRNA was significantly enriched in LIN28B knockdown cells (Fig. 5B). Conversely, overexpression of LIN28B led to a marked reduction of RPL26-bound *TP53* mRNA (Fig. 5C).

We next sought to determine whether the competition between LIN28B and RPL26 was functional. Using a luciferase reporter containing the *TP53* 5′UTR mRNA sequence, we found that overexpression of RPL26 significantly elevated the luciferase activity in HeLa cells compared with controls (Fig. 5D). However, overexpression of LIN28B blocked the enhancing effect of RPL26 on luciferase activity. In addition, we detected p53 protein expression in response to LIN28B and RPL26 co-transfection by western blot analysis and found that RPL26-mediated enhancement of p53 protein was moderately inhibited by LIN28B expression (Fig. 5E). These data demonstrate that LIN28B not only competes with RPL26 at the 5′UTR of *TP53* mRNA but also suppresses the enhancer function of RPL26 in regulating p53 translation.

### LIN28B inhibition sensitizes cells to p53–MDM2 inhibitors

**Fig. 6:**
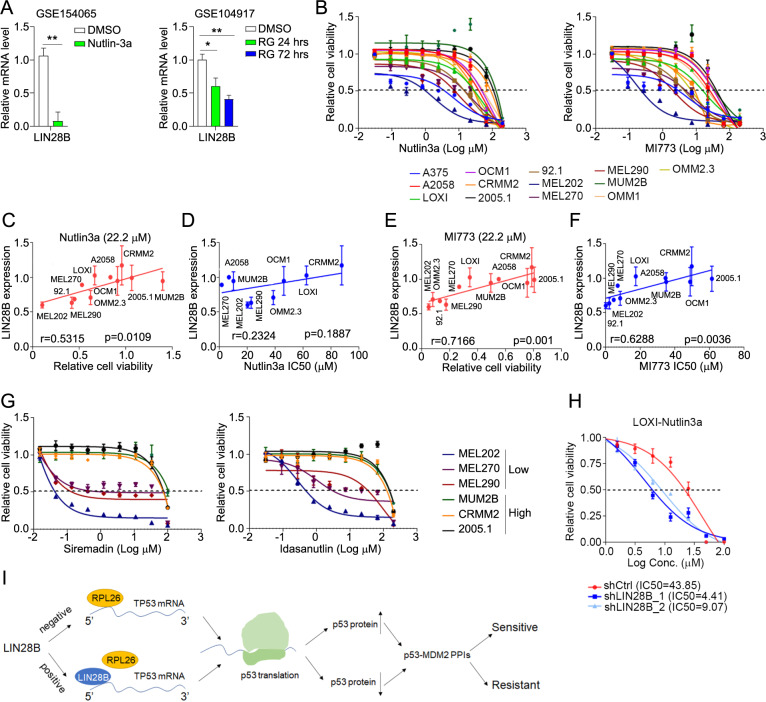
LIN28B inhibition sensitizes cells to p53–MDM2 inhibitors. **A** Relative mRNA level of LIN28B in control (DMSO) and p53–MDM2 inhibitor Nutlin3a, RG7388 (RG, Idasanutlin) treated cells by analyzing the GSE154065 (HCT116) and GSE104917 (NB1691 neuroblastoma) datasets. *p < 0.05, **p < 0.01. **B** Relative cell viability as determined by CCK8 in ocular melanoma cells treated by various concentrations of Nutlin3a and MI773. **C** Correlation between relative cell viability and LIN28B protein level in ocular melanoma cells treated by Nutlin3a (22.2 μM). **D** Correlation between Nutlin3a IC50 and LIN28B protein level in ocular melanoma cells. **E** Correlation between relative cell viability and LIN28B protein level in ocular melanoma cells treated by MI773 (22.2 μM). **F** Correlation between MI773 IC_50_ and LIN28B protein level in ocular melanoma cells treated. **G** Relative cell viability as determined by CCK8 in ocular melanoma cells treated by various concentrations of Siremadin and Idasanutlin (RG7388). Representative data of three independent experiments (mean ± s.e.m.). **H** Relative cell viability of control (shCtrl) and knockdown (shLIN28B) LOXI cells treated by various concentrations of Nutlin3a. The IC_50_ of each group was shown. Representative data of three independent experiments (mean ± s.e.m.). **I** Schematic diagram of the working model.

It remains a question whether our findings can be therapeutically exploited. Notably, LIN28B was significantly down regulated in cancer cells under the treatment of Nutlin3a (GSE154065) [35] and RG7388 (GSE104917) [36], two established p53–MDM2 PPIs (Fig. 6A). Further studies in ocular melanoma cells demonstrated the positive correlation between LIN28B expression level and cell viability under the treatment of p53–MDM2 PPI Nutlin3a and MI773 (Fig. 6B, C and E). Consistently, the IC50s of Nutlin3a and MI773 showed similar trends toward LIN28B expression (Fig. 6D, F). We further confirmed these results using another two types of p53–MDM2 PPIs, Siremadin and Idasanutlin (RG7388), in LIN28B high- and low-expressing cells (Fig. 6G). In addition, knockdown of LIN28B rendered LOXI cells sensitive to Nutlin3a treatment as exhibited by the remarkable reduction of IC50s (Fig. 6H). To evaluate the contribution of let-7 to LIN28B-mediated sensitivity to p53–MDM2 PPIs, we used the LIN28-let-7 antagonist HY-100692 to unleash the suppression of let-7 by LIN28A/B [37]. Using a concentration significantly higher than the reported IC_50_ of HY-100692, we found that LIN28B-mediated sensitivity to MI773 remained unchanged (Supplementary Fig. 7A, B), suggesting a negligible role of let-7. These findings suggest the potential of LIN28B as a stratification biomarker to the therapy of p53–MDM2 PPIs.

## Discussion

In this study, we have highlighted a molecular mechanism that links the oncogenic protein LIN28B to p53 regulation. LIN28B preferentially binds to the 5′UTR of *TP53* mRNA, competing with the p53 translation enhancer RPL26 and consequently inhibiting *TP53* mRNA translation (Fig. 6I). Reportedly, the DNA-binding activity of p53 can be compromised by the R175H mutation, however the transcriptional and proapoptotic functions of p53 can be reactivated under certain circumstances [38, 39]. Our results in p53-mutant TOV-112D (R175H) cells indicated that the R175H mutation did not affect the translational suppression of LIN28B on p53.

Blocking let-7 maturation by LIN28B is well established, but LIN28B also recognizes and directly binds to the “GGAGA” motifs within certain mRNAs and affect their translation, thereby regulating the cell cycle, mRNA metabolism, glycolysis, and other cellular processes [27, 40, 41]. By analyzing the secondary structure of *TP53* mRNA, we observed typical “GGAG” motifs in the *TP53* 5′UTR, which suggested that *TP53* mRNA is a canonical target of LIN28B. In the analyzed eCLIP dataset, direct binding to the TP53 5′UTR was detected at the physiological LIN28B expression level, which also suggested the 5′UTR region contains a high-affinity binding site. Let-7 overexpression significantly increased the apoptosis induced by camptothecin, but did not increase p53 protein level. Thus, the let-7 circuit was likely negligeable to LIN28B-mediated p53 suppression. Previous works reported that LIN28 may influence the translation of key factor mRNAs such as IGF-2 to enhance its translation efficiency [42–44]. Endoplasmic reticulum (ER)-localizing LIN28A could serve as a global repressor of the secretory pathway by inhibiting the translation of ER-designated mRNAs and reducing the synthesis of ER or Golgi lumen proteins [45]. Nevertheless, the competitive mechanism of action between LIN28B and RPL26 at the 5′UTR of *TP53* mRNA has not been reported previously. These results have provided a framework to understand the general mechanism of translational modulation by LIN28B.

Our study also supports the notion that therapeutic strategies aiming to reinstate p53 function via inhibiting the protein–protein interaction between p53–MDM2 are less effective in the scenario that cancer cells have already adopted mechanisms beyond protein degradation to derail p53 signaling. The efficacies of PPIs targeting p53–MDM2 are currently being tested clinically [12, 46]. Our results highlighted that LIN28B may be used as biomarker to predict the therapeutic responses. In this regard, it can be crucial to examine LIN28B levels before conducting therapies using anti-p53 degradation inhibitors. These findings further advance our knowledge of the regulatory circuitry of how LIN28B modulates p53, which is important for developing novel ways to treat cancers with p53 dysfunction.

## Materials and methods

### Cell culture

Ovarian cancer cell lines were purchased from the American Type Culture Collection (ATCC) and the Division of Cancer Treatment and Diagnosis (DCTD) Tumor/Cell Line Repository and were cultured in RPMI1640 (Invitrogen, CA, USA) supplemented with 10% fetal bovine serum (FBS) (Gibco, CA, USA). OCM1 and OCM431 cells were kindly provided by Dr. John F. Marshall (Cancer Research UK Clinical Center, John Vane Science Centre, London, UK) and were maintained in complete Dulbecco’s modified Eagle’s medium (DMEM, Gibco) supplemented with 10% FBS. Ocular melanoma cells were kindly provided by Dr. Martine Jager (Leiden University, Netherlands). CRMM1 and CRMM2 cells were maintained in DMEM/F12 (Gibco) supplemented with 10% FBS. A375, A2058, 293T and LOXI cells were maintained in DMEM (Gibco) supplemented with 10% FBS. MUM2B, OMM2.3, MEL285, and MEL290 cells and PIG1 control cells were maintained in RPMI1640 supplemented with 10% FBS. All cell cultures were supplemented with 1% penicillin/streptomycin (Gibco) and maintained at 37 °C in a 5% CO_2_ humidified atmosphere. The p53 status of cell lines was listed in Supplementary Table 1.

### RNA interference (RNAi)

siRNA and control oligonucleotides were purchased from IDT. Transfections were performed using the Lipofectamine™ RNAiMAX transfection reagent (Invitrogen) following the manufacturer’s instructions. Cells were incubated in the media containing the transfection mixture for 72 h before harvesting.

### Virus transduction and generation of stable cell lines

Two lentiviral shRNAs targeting human LIN28B (pLKO.1, TRCN0000122191; TRCN0000122599) were purchased from Open Biosystems. Non-targeting shRNA (SHC002) was used as a control. For the construction of knock out cell lines, The cas9, sgCtrl (PGMLV-GM1, ACGGAGGCTAAGCGTCGCAA) and LIN28B sgRNA (PGMLV-GM1, #1ACATCGACTGGAATATCCAA; #2 CATCGACTGGAATATCCAAG) lentiviruses were purchased from Genomeditech (China). Lentiviral transfection was conducted according to the manufacturer’s instructions. Blasticidin and puromycin were used to select the resistant cells. Mutation of the targeted gene was identified by gene sequencing. For studies using the constructed pMSCV-neo-hLIN28B retroviral expression vector, the pMSCV-neo empty vector was used as a control. The lentiviral vector and packaging vectors were transfected into the packaging cell line 293T using the FuGene6 Transfection Reagent (Roche). Retroviral vectors were transfected into the packing cell line PT67 (Clontech) using the FuGene6 Transfection Reagent. The medium was changed 8 h post-transfection and the medium containing the virus was collected 48 h later. Targeted cells were infected with lentivirus in the presence of 8 µg/ml polybrene (Sigma). The GFP-RPL26 plasmid was purchased from Addgene (Plasmid #31980). For CRISPR-Cas9 knockout, the human LIN28B sgRNA (TTCTCAGGCGGGGCTAGCAA) was purchased from Genomeditech. Efficacy of LIN28B knockdown or knockout were determined by western blots.

### RNA extraction and real-time PCR

Total RNA was isolated from 100 to 500 mg frozen tissue or 1 × 10^6^ cultured cells using TRIzol reagent (Invitrogen). The quality and quantity of the isolated RNA were analyzed using a Bioanalyzer 2100 system (Agilent). Total RNA was reverse-transcribed using the high-capacity RNA-to-cDNA Kit (Applied Biosystems) according to the manufacturer’s instructions. cDNA was quantified by real-time PCR on an ABI Prism 7900 Sequence Detection System (Applied Biosystems). PCR was performed using SYBR Green PCR Core reagents (Applied Biosystems) according to the manufacturer’s instructions. PCR amplification of the housekeeping gene GAPDH or 18S ribosomal RNA gene (18S) was performed for each sample as control for sample loading and for normalization across samples. The PCR primer sequences are as follows: *TP53: CAAAGAAGAAACCACTGGATGGA* (forward), *CTCATTCAGCTCTCGGAACATCT* (reverse); *LIN28B: AAGAAGACCCAAAGGGAAGACAC* (forward), *CACTTCTTTGGCTGAGGAGGTAG* (reverse); *18S: AAACGGCTACCACATCCAAG* (forward), *CCTCCAATGGATCCTCGTTA* (reverse); *GAPDH: ACACCATGGGGAAGGTGAAG* (forward), *AAGGGGTCATTGATGGCAAC* (reverse); TP53 5′UTR primer #1: *AAGTCTAGAGCCACCGTCCA* (forward), *GTGTCACCGTCGTGGAAAG* (reverse); *TP53* 5′UTR primer #2: *CCTCCCATGTGCTCAAGACT* (forward), *GTGTCACCGTCGTGGAAAG* (reverse); *BAX: TGCTTCAGGGTTTCATCCAG (forward)*, *GGCGGCAATCATCCTCTG (reverse)*; *GADD45: TCAGCGCACGATCACTGTC* (forward), *CCAGCAGGCA CAACACCAC (reverse)*; *CDKN1A: CCTCATCCCGTGTTCTCCTTT* (forward), *GTACCACCCAGCGGACAAGT (reverse)*; and *HMGA2: GCAGAAGCCACTGGAGAAAAAC* (forward), *GAGCAGGCTTCTTCTGAACAACT (reverse)*.

### RNA-immunoprecipitation (RNA-IP)

Cells (5 × 10^6^) were lysed for 15 min on ice in a polysome lysis buffer containing 100 mM KCl, 5 mM MgCl_2_, 10 mM HEPES pH 7.0, 0.5% NP-40 detergent supplemented with fresh 1 mM dithiothreitol (DTT), 100 U/ml RNase Out (Invitrogen), 400 µM vanadyl-ribonucleoside complex (VRC) (New England Biolabs), and a protease inhibitor cocktail (Sigma). The cell lysate was further diluted (1:10) with NT2 buffer containing 50 mM Tris–HCl pH 7.4, 150 mM NaCl, 1 mM MgCl_2_, 0.05% NP-40 supplemented with fresh 200 U/ml RNase Out, 400 µM VRC, 1 mM DTT, 20 mM EDTA, and a protease inhibitor cocktail. The insoluble particles in the lysate were removed by centrifugation at 15,000 × *g* for 15 min at 4 °C. LIN28B antibody (1:75, Cell Signaling Technology), RPL26 antibody (1:50, Abcam) or control IgG was added to protein-A Sepharose beads (Sigma) that had been pre-incubated in 5% bovine serum albumin (BSA)–NT2 buffer for 1 h at 4 °C. After gentle rotation for 4 h at 4 °C, the beads were washed four times in cold NT2 buffer and added to the cell lysates (10 µl beads/ml lysate). Immunoprecipitation was performed by gentle rotation overnight at 4 °C. The immunoprecipitated complexes were washed four times in NT2 buffer and resuspended in 100 µl NT2 buffer containing 30 µg proteinase K (QIAGEN) to release the RNP complex. TRIzol reagent was used to extract RNA from the immunoprecipitation.

### Annexin V apoptosis assays

Annexin-V staining was performed using an apoptosis detection kit (R&D Systems). Both adherent and non-adherent cells were collected, washed with PBS, and resuspended in binding buffer containing 10 mM HEPES (pH 7.4), 140 mM NaCl, and 2.5 mM CaCl_2_. After 15 min incubation of Annexin-V-FITC antibody at room temperature, cells were analyzed using a FACScan flow cytometer (Becton Dickinson).

### Western blot

Cells were lysed in mammalian protein extraction reagent (Pierce). Protein concentrations were quantified using a bicinchoninic acid (BCA) protein assay kit (Pierce), and equal amounts of proteins (30 µg) were separated by 10% SDS–PAGE under denaturing conditions and transferred to PVDF membranes (Millipore). Membranes were blocked in 5% non-fat milk (Bio-Rad) and then incubated with primary antibodies, followed by incubation with a secondary antibody conjugated with horseradish peroxidase (HRP, 1:10,000; Amersham Biosciences). Immunoreactive proteins were visualized using the LumiGLO chemiluminescent substrate (Cell Signaling Technology). The primary antibodies are as follows: β-Actin (1:10,000; Sigma); α-Tubulin (1:5000; Sigma); LIN28B (1:1000; Cell Signaling Technology, #11965); TP53 (1:1000; Cell Signaling Technology, #2527); RPL26 (1:1000; Abcam, ab59567); cleaved caspase-3 (1:1000; Cell Signaling Technology, #9664); Nucleolin (1:1000; Cell Signaling Technology, #14574); MDM2 (1:1000; Cell Signaling Technology, #51541); MDM4 (1:1000; Sigma-Aldrich, 04-1556); and LaminB1 (1:1000; Abcam, ab16048).

### Caspase-3/7 activity assay

Caspase-3/7 activity assays were performed using the Apo-ONE Homogeneous Caspase-3/7 Assay kit (Promega) following the manufacturer’s instructions. Cells were plated in 96-well plates and four to six wells were assayed for each sample. Experiments were repeated twice. The resulting fluorescent intensity was quantified using a Fluoroskan Ascent FL microplate reader (Thermo Scientific).

### Cell viability assay

Cell viability was detected by CCK8 assay (C6030, New Cell & Molecular Biotech Co., Ltd). Briefly, cells were seeded in 96-well plates with a density of 2 × 10^3^ cells/well. After treatments, CCK8 was added to the cell culture medium and maintained for additional 1 h. The absorbance was determined at 450 nm by a microplate reader (BioTek). Nutlin-3a (HY-10029), Siremadlin (HY-18658), Idasanutlin (HY-15676), and HY-100692 were purchased from MCE MedChemExpress. MI773 (S7649) was purchased from Selleck.

### Immunohistochemistry

Immunohistochemistry was performed using the VECTASTAIN ABC Kit (Vector). The following primary antibodies were used in this study: rabbit anti-human LIN28B (1:400) and mouse anti-human p53 (1:1800) (both from Cell Signaling Technology). Antibodies were incubated overnight at 4 °C and the immunoreaction was visualized using 3,3′-diaminobenzidine. Images were collected and analyzed using Image-Pro Plus software (Media Cybernetics).

### RNA stability

The decay rate of *TP53* mRNA was measured using a real-time PCR-based time-course analysis. Briefly, actinomycin D (10 µg/ml, Sigma) was added to the culture medium to block transcription. At 0, 3, 6, 12, and 18 h post-actinomycin D treatment, cells were harvested and total RNA was extracted. Real-time PCR was performed to determine the levels of *TP53* mRNA. The mRNA decay curve was plotted by setting the level at 0 h as 100%.

### Protein stability

To measure the half-life of endogenous p53, cells were treated with 100 μg/ml cycloheximide (Sigma) and harvested at 0, 2, 4, 8, and 12 h post-cycloheximide treatment. The p53 protein level was detected by western blot and the protein decay curve was plotted by setting the protein level at 0 h as 100%.

### RNA-pull down assay

The *TP53* 5′UTR was amplified from cDNAs by PCR and sub-cloned into the pBluescriptII SK+ vector. Biotin-labeled *TP53* 5′UTR RNA was prepared using the Biotin RNA Labeling Mix (Roche) and T7 RNA polymerase (Stratagene) following the manufacturer’s instructions. Biotinylated RNAs were treated with RNase-free DNase I and further purified on G-50 Sephadex Quick Spin columns (Roche). Biotinylated RNA (2 µg) was mixed with 1 mg of pre-cleared, transcription and splicing-competent A2780 whole cell lysate in RNA–IP buffer supplemented with tRNA (100 µg/µl). Samples were incubated overnight at 4 °C with gentle rotation. Whole cell lysate (50 µg) was used as input. Washed streptavidin agarose beads (Invitrogen) (60 µl) were added to each binding reaction and samples were further incubated at 4 °C for 1 h. Beads were washed five times with NT2 buffer by gentle tapping and brief centrifugation, followed by boiling in SDS protein-loading buffer. The eluted protein and input samples were examined by western blot analysis.

### ^35^S metabolic labeling

Cells were pre-incubated in RPMI1640 (without methionine and cysteine) that was supplemented with 10% dialyzed FBS for 1 h. Cells were then labeled with 100 μCi/ml of ^35^S methionine for 20 min. Cells were washed with PBS, lysed in buffer (50 mM Tris–HCl, pH 7.4, 150 mM NaCl, 1 mM EDTA, and 1% TRITON X-100), and incubated at 4 °C for 30 min with gentle shaking. The insoluble fraction was removed from the cell lysate by centrifugation at 13,000 × *g* for 20 min and the protein concentration was measured using the BCA assay. Whole cell lysate (50 µg) was used as input. Whole cell lysate (1 mg) was used for immunoprecipitation following standard protocols. Briefly, protein A/G-PLUS agarose beads (Sigma) were blocked with 5% BSA for 1 h, and the beads were coated with a mouse monoclonal antibody against p53 (#2524, Cell Signaling Technology, 1:50) by incubation for 4 h at 4 °C. Cell lysates were incubated with coated beads at 4 °C with overnight rotation. The beads were collected by centrifugation and washed extensively with lysis buffer. Immunoprecipitated complexes were boiled in SDS sample buffer and resolved by gel electrophoresis, followed by transfer to nitrocellulose membranes. The total amount of immunoprecipitated TP53 on the membrane was detected using an autoradiography approach.

### *TP53* response element reporter assay

Cells (1 × 10^4^) were plated onto 24-well plates. After incubation overnight, cells were transiently transfected with firefly luciferase reporter plasmids containing a p53 *cis*-acting enhancer element (pTA-p53, 100 ng/well), a TP53 5′UTR (pGL3, 100 ng/well) or a 3′UTR response element, (pmirGLO, 100 ng/well) together with LIN28B cDNA (250 ng/well), GFP-RPL26 cDNA (250 ng/well) or LIN28B siRNA oligonucleotides (30 nM). The Renilla luciferase reporter plasmid (pRL; 10 ng/well) was co-transfected to quantitate the transfection efficiency. Cells were harvested 48 h post-transfection, and firefly and Renilla luciferase were measured sequentially in a Fluoroskan Ascent FL microplate reader (Thermo Scientific). TP53 5′UTR and 3′UTR sequences used for reporter assay were listed in Supplementary Table 2.

### Statistical analysis

Statistical analyses were performed using GraphPad Prism 8 software. Unpaired *t*-tests were used to generate two-tailed *p* values and *p* < 0.05 indicated statistical significance.

## Supplementary information

Supplementary Figure

Supplementary Table 1

Supplementary Table 2

## Data Availability

All data needed to evaluate the conclusions in the paper are present in the paper and/or the Supplementary Materials. The eCLIP-seq dataset was downloaded from GEO database under accession code GSE178259. Additional data related to this paper may be requested from the authors.
