# Supplementary material for: LIN28B inhibition sensitizes cells to p53-restoring PPI therapy through unleashed translational suppression
Source: Oncogenesis. 2022 Jul 2;11(1):37. doi: 10.1038/s41389-022-00412-8 (PMC9250532; doi:10.1038/s41389-022-00412-8)
Supplement: Supplementary file 1 — Supplementary Figure [file 41389_2022_412_MOESM1_ESM.docx]

**Supplementary Materials**


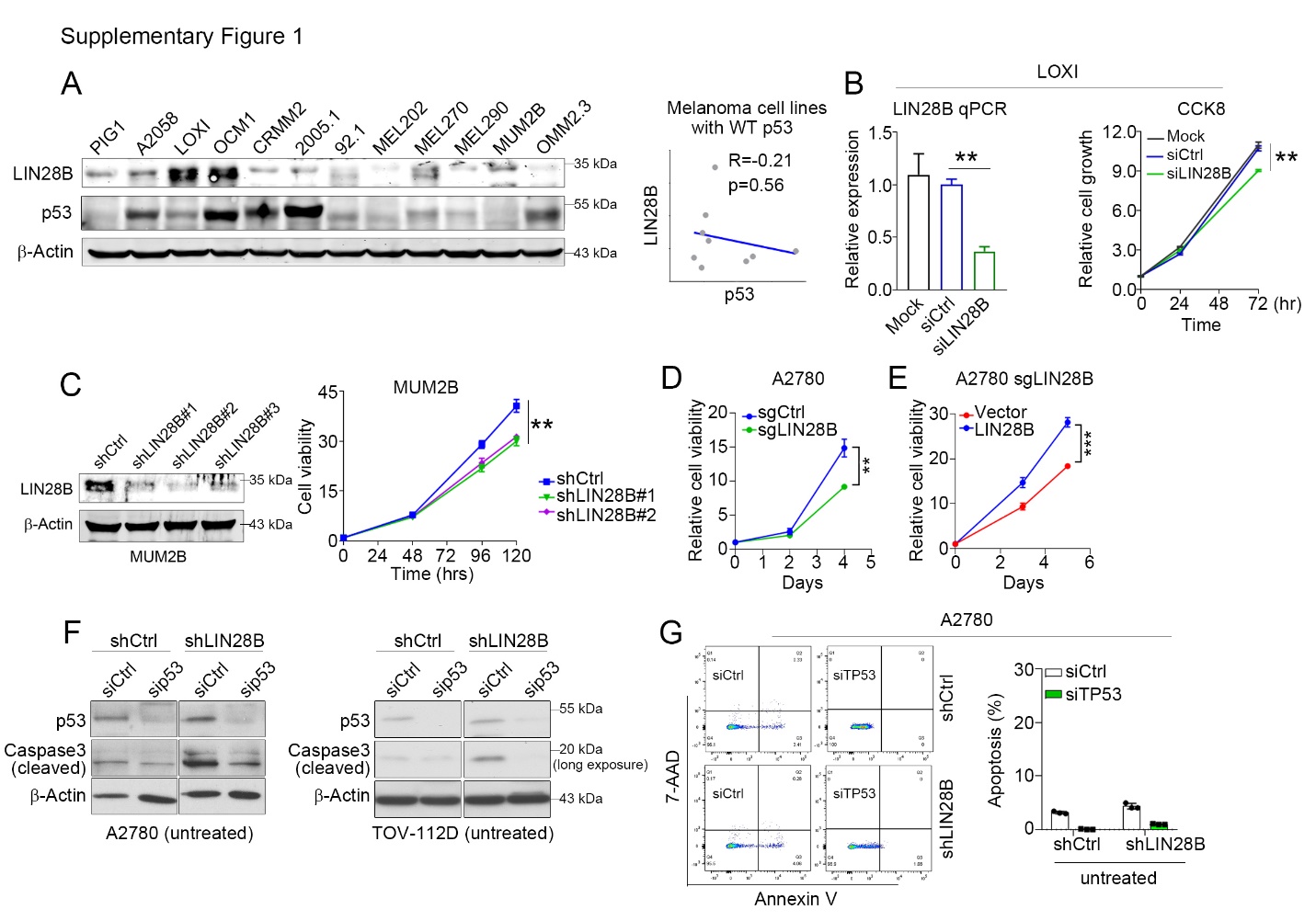


**Supplementary Figure 1. LIN28B is activated in ocular melanoma cancer cell lines and promotes cell growth.**

**A,** Western blots of LIN28B and p53 expression in ocular melanoma cancer cell lines (Left). Correlation of LIN28B and p53 protein expression in p53 wild-type (WT) melanoma cancer cell lines (Right). **B,** qPCR and cell growth assay in control (Mock, siCtrl) and LIN28B-depleted (siLIN28B) LOXI cells. **p<0.01. Representative data of three independent experiments (mean ± s.e.m.). **C,** Western blots and cell growth assay in control (shCtrl) and LIN28B-depleted (shLIN28B) MUM2B cells. **p<0.01. Representative data of three independent experiments (mean ± s.e.m.). **D,** Cell growth assay in control CRISPR (sgCtrl) and LIN28B CRISPR (sgLIN28B) A2780 cells. **p<0.01. Representative data of three independent experiments (mean ± s.e.m.). **E,** Cell growth assay in Vector- and LIN28B-overexpressing A2780 cells with LIN28B depletion (sgLIN28B). ***p<0.001. Representative data of three independent experiments (mean ± s.e.m.). **F,** Western blots of p53 and cleaved caspase-3 in control (si*Ctrl*) and p53 knockdown (si*TP53*) cells without treatment. **G**, FACS analysis of apoptosis (Annexin-V/PI labeling) in control (si*Ctrl*) and p53 knockdown (si*TP53*) cells without treatment. Representative data of three independent experiments (mean ± s.e.m.).

**
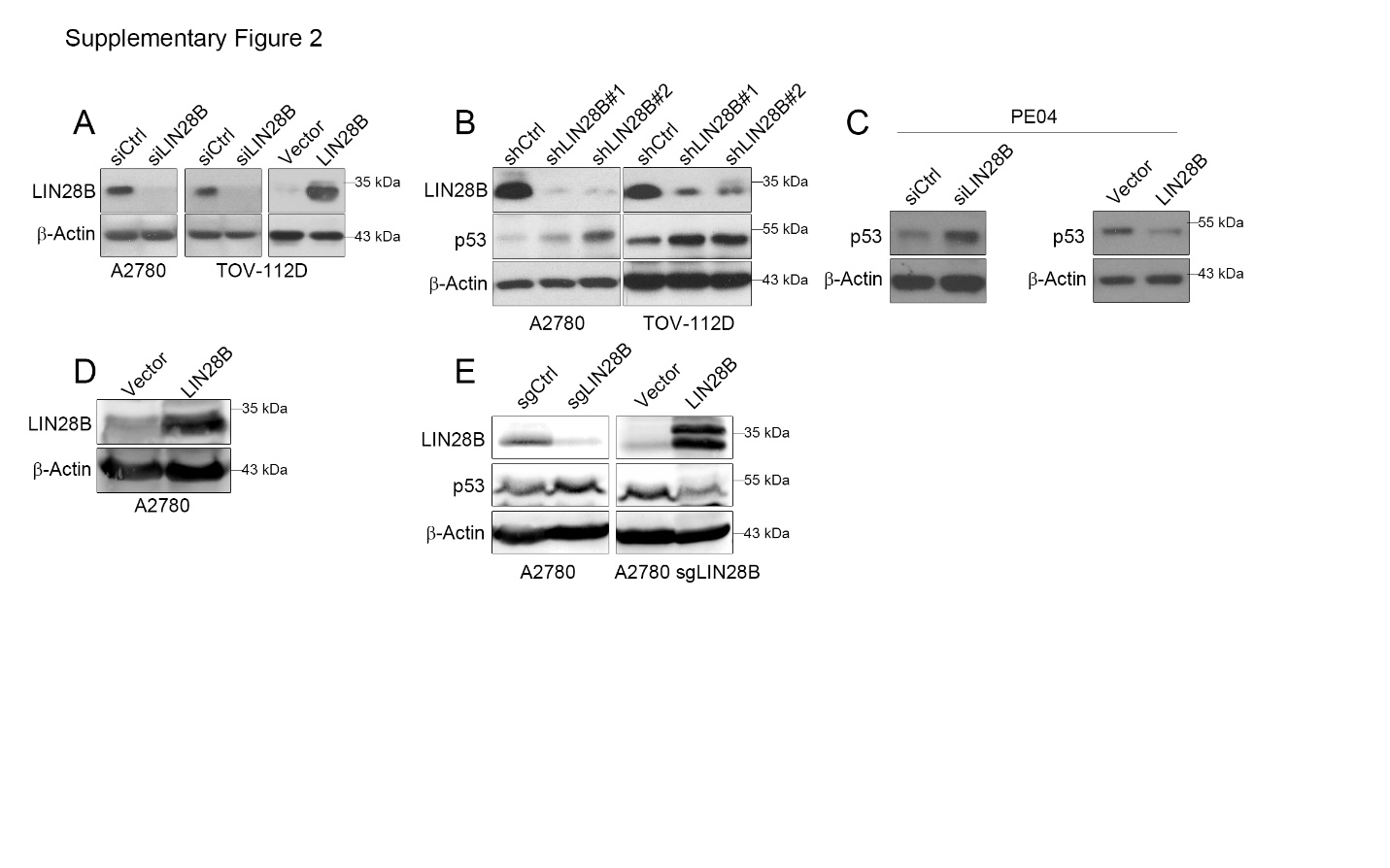
**

**Supplementary Figure 2. LIN28B suppresses p53 protein level.**

**A,** Representative western blots of LIN28B knockdown and overexpression efficiency in A2780 and TOV-112D cells. **B**, Representative western blots of LIN28B and p53 in control (shCtrl) and LIN28B knockdown (shLIN28B) A2780 and TOV-112D cells. **C**, Representative western blots of p53 in PE04 cells with LIN28B knockdown (siLIN28B) and overexpression (LIN28B). **D**, Representative western blots of LIN28B in A2780 cells with control (Vector) and overexpression (LIN28B). **E**, Representative western blots of LIN28B and p53 in control CRISPR (sgCtrl) or LIN28B CRISPR (sgLIN28B) A2780 cells, and Vector- or LIN28B-overexpressing A2780 cells with LIN28B depletion (sgLIN28B).

**Supplementary Figure 3. Basal level of p53 in ovarian and breast cancer cell lines.**


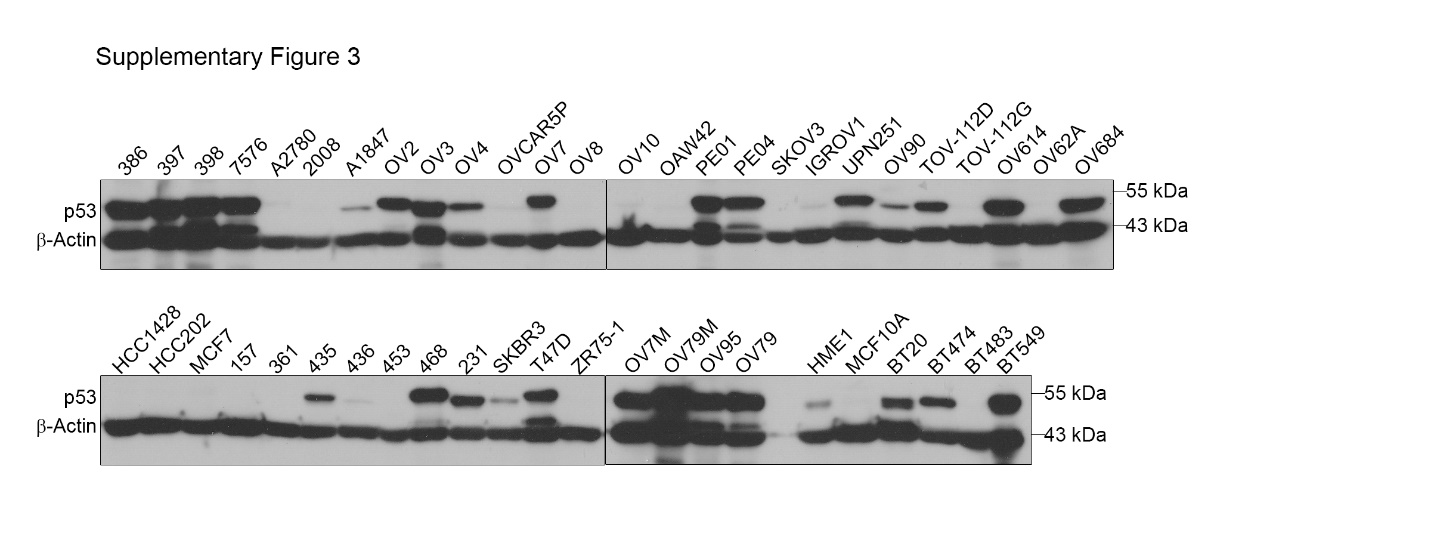


Western blots of p53 expression in ovarian and breast cancer cell lines related to Figure 2C.

**
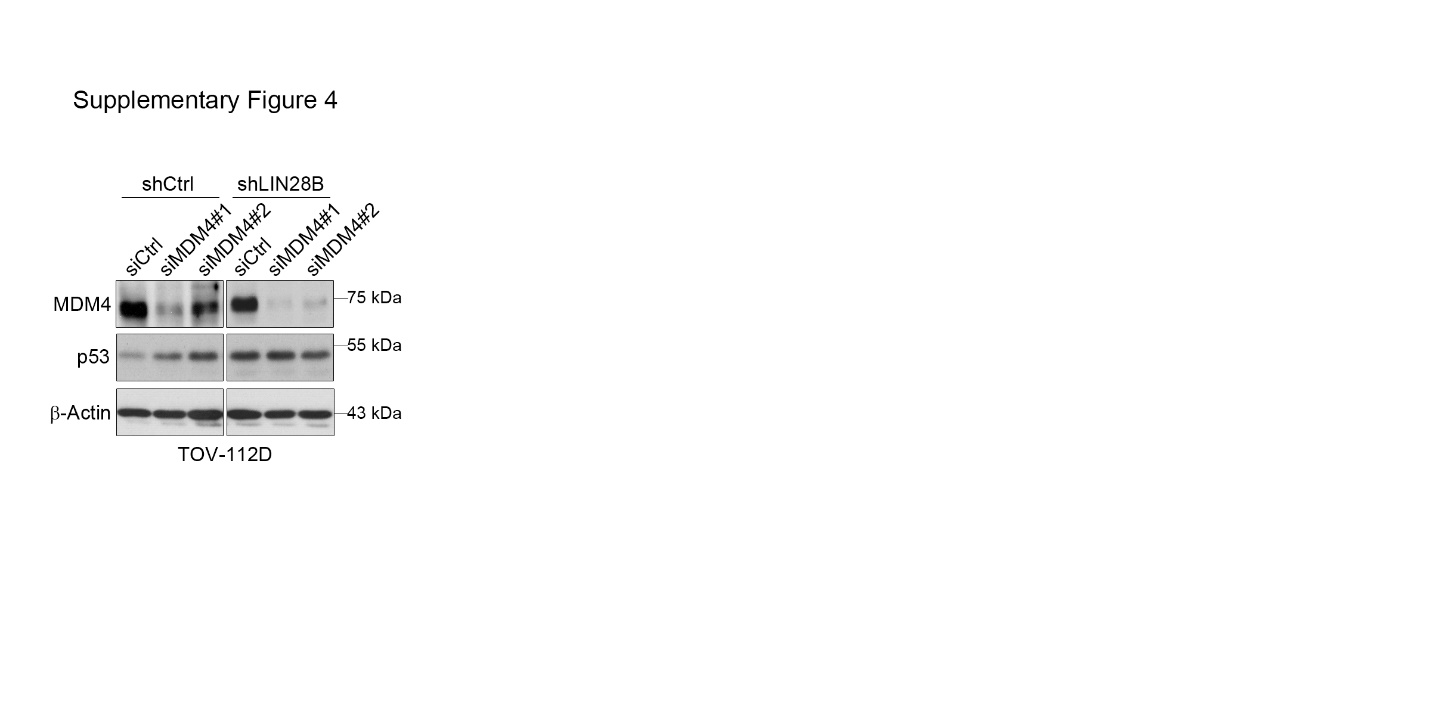
**

**Supplementary Figure 4. Inhibition of MDM4 does not further increase p53 protein level under LIN28B knockdown.**

Western blots of MDM4 and p53 in control (shCtrl) and LIN28B knockdown (shLIN28B) TOV-112D cells transfected with control (siCtrl) or siRNA targeting MDM4 (siMDM4#1, #2) for 48 h.


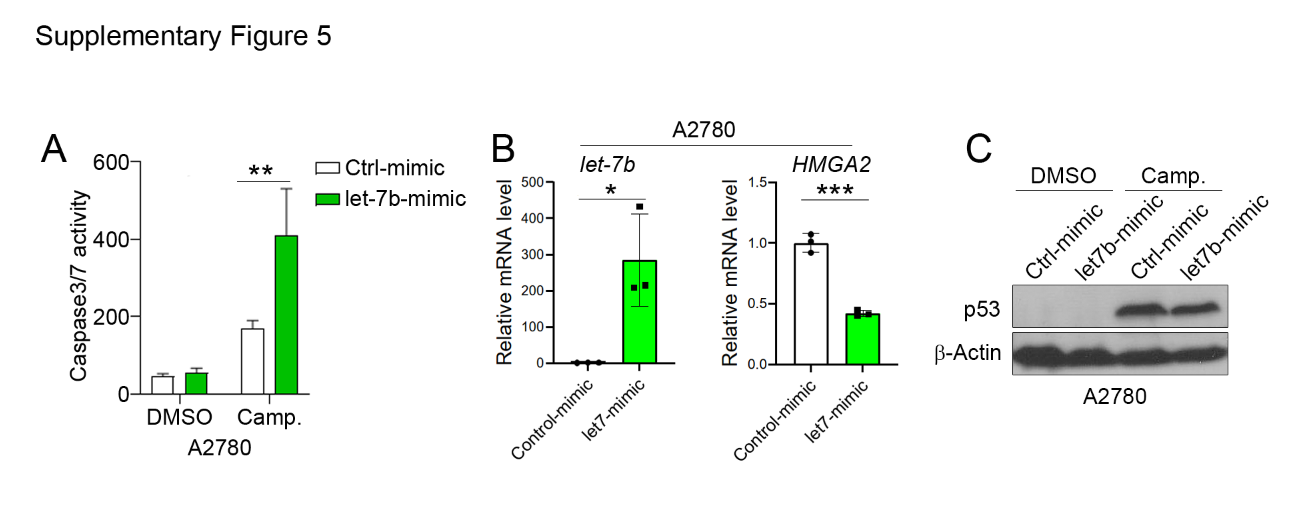


**Supplementary Figure 5. MicroRNA let-7b overexpression does not affect p53 protein level.**

**A,** Caspase-3/7 activity in A2780 cells transfected with control (Ctrl-mimic) and microRNA let-7b (Let7b-mimic) (30 nM) for 48 h. Camp., camptothecin (2 μM, 8 h). Representative data of three independent experiments (mean ± s.e.m.). **p<0.01 **B**, qPCR of *HMGA2* and *let-7b* in A2780 cells transfected with control (Ctrl-mimic) and microRNA let-7b (Let7b-mimic) (30 nM) for 48 h. *p<0.05, ***p<0.001. Representative data of three independent experiments (mean ± s.e.m.). **C,** Representative western blots of p53 in A2780 cells transfected with control (Ctrl-mimic) and microRNA let-7b (let-7b-mimic) (30 nM) for 48 h. Camp., camptothecin (2 μM, 8 h).


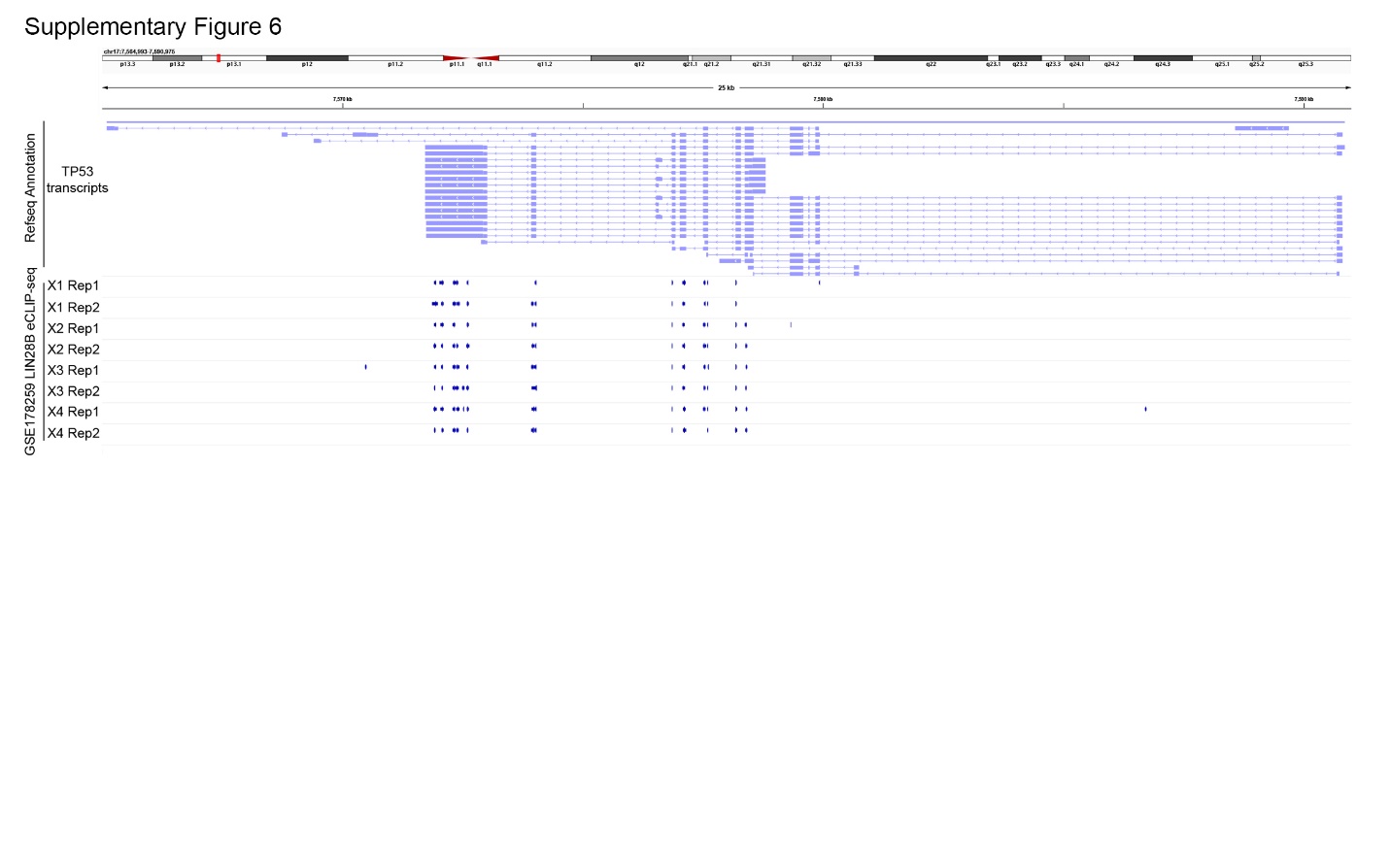


**Supplementary Figure 6. LIN28B eCLIP-seq detects p53 mRNA binding.**

The LIN28B eCLIP-seq dataset performed in 293T (GSE178259) was analyzed. Cell lines of different LIN28B expression levels from the physiological level to the 7-fold level were labelled as X1 to X4. Each cell line had two biological replicates.


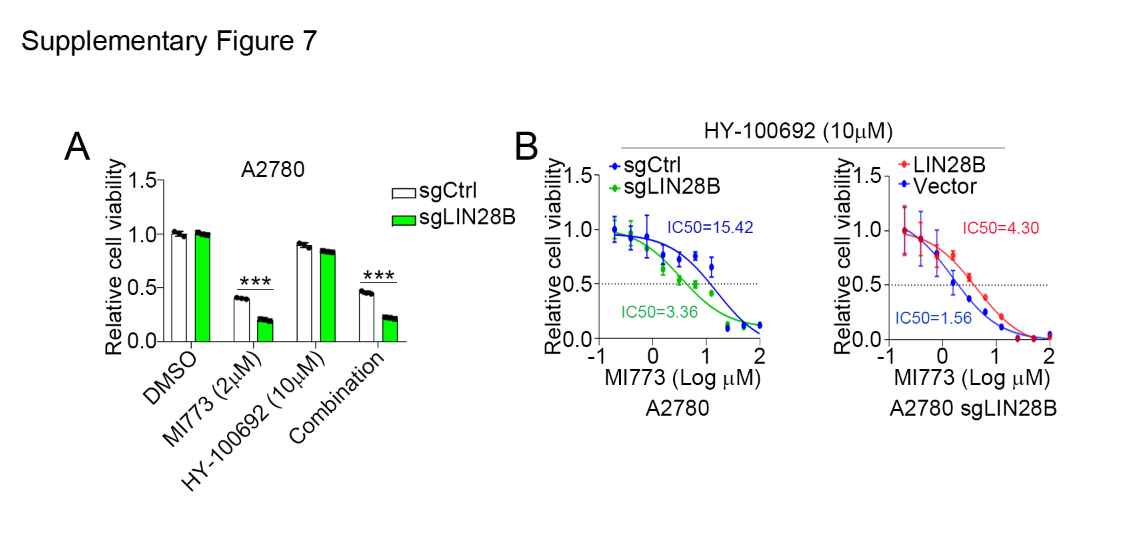


**Supplementary Figure 7. HY-100692 does not affect LIN28B-mediated sensitivity to MI773.**

**A,** Cell growth assay in control (sgCtrl) and LIN28B-depleted (sgLIN28B) A2780 cells treated by MI773 (2 μM), HY-100692 (10 μM) and combination (MI773+HY-100692). ***p<0.001. Representative data of three independent experiments (mean ± s.e.m.). **B,** IC50 assessment by cell growth assay in A2780 cells under MI773 and HY-100692 treatment.
